# Anti-Rheumatic Drugs May Ameliorate the Clinical Course and Outcome of COVID-19 In Rheumatoid Arthritis Patients

**DOI:** 10.31138/mjr.33.1.68

**Published:** 2022-03-31

**Authors:** Eleftherios Pelechas, Vassiliki Drossou, Paraskevi V. Voulgari, Alexandros A. Drosos

**Affiliations:** Rheumatology Clinic, Department of Internal Medicine, Medical School, University of Ioannina, Ioannina, Greece

**Keywords:** COVID-19, rheumatoid arthritis, hydroxychloroquine, tocilizumab, csDMARDs, bDMARDs

## Abstract

Current data demonstrated that in patients with coronavirus disease-19 (COVID-19), there is a dysregulation of the immune system during the severe form of the disease. This dysregulation is expressed with an uncontrolled release of pro-inflammatory cytokines such as interleukin-1 (IL-1), IL-6, IL-17, tumour necrosis factor alpha (TNFa) and chemokines, associated with increased serum ferritin levels and other acute phase reactants. On the other side, these cytokines play a pivotal role in autoimmune rheumatic diseases (ARD), mostly in rheumatoid arthritis (RA) and the spondyloarthropathies. Patients affected with ARD represent a particular vulnerable group, considering that they may be in an immunocompromised status due to their ailment and its treatment on one side, but on the other side, they may be protected from their immunosuppressive therapy. To this end, we present five patients with RA treated with conventional synthetic (cs) disease-modifying anti-rheumatic drugs (DMARDs) and biologic (b) DMARDs who were affected from COVID-19 and we will try to give answers to the above hypothesis.

The pandemic of coronavirus disease 19 (COVID-19) caused by the severe acute respiratory syndrome corona-virus-2 (SARS-CoV-2) is a matter of concern worldwide. Environmental factors such as smoking, obesity, cardio-respiratory diseases, diabetes mellitus and age may increase the severity of COVID-19.^1,2^ Current data demonstrated an acute dysregulation of the immune system during the severe form of the disease. The above is demonstrated by the clinical picture and laboratory findings, suggesting a severe inflammatory response of the innate immunity, affecting mainly the respiratory system with the recruitment in the lung parenchyma of macrophages, monocytes, and lymphocytes, followed by the thrombotic diathesis and multiorgan failure. Beside the clinical manifestations, the immune response is expressed with the activation of macrophages and lymphocytes generating a plethora of cytokines like interleukin (IL)-1, IL-6, IL-17, tumour necrosis factor a (TNFa), and various chemokines. As a consequence, high levels of acute phase reactants are produced, such as C-reactive protein (CRP), hyperferritinaemia and hyper-fibrinogenaemia. Afterwards, a dysregulation of the adaptive immunity with reduction of lymphocytes mostly CD4^+^ and CD8^+^ T-cells takes place.^3,4^

On the other hand, autoimmune rheumatic diseases (ARD) are also characterised by a dysregulation of the immune system in which several pro-inflammatory cytokines such as IL-1, IL-6, IL-17, and TNFa which are involved in the pathogenesis of rheumatoid arthritis (RA), the spondyloarthropathies (SpA), and inflammatory bowel disease (IBM).^5^ In the last two decades the introduction of biological (b) disease-modifying anti-rheumatic drugs (DMARDs) targeting cytokines, T and B cells, and with the use of conventional synthetic (cs) DMARDS, has revolutionized the treatment of the above diseases.^6,7^

The questions which arise here are: what is the impact of COVID-19 inflection in patients with ARD “already treated with cs and bDMARDs”? Does their immunocompromised status due to the disease itself and its treatment put their life in danger? Are they protected against COVID-19 infection due to long use of cs and bDMARDs?.^8^ For this reason, we present five patients with RA, treated with cs and bDMARDs, who were infected with COVID-19, and discuss the relevant literature trying to answer the above questions. An informed consent has been obtained from all patients.

## CASE PRESENTATION 1

A 42-year-old woman was diagnosed 6 years earlier with RA on the basis of clinical and laboratory findings. More specifically, bilateral symmetrical polyarthritis of the small joints of the hands, positive anti-citrullinated protein antibodies (ACPA), positive IgM rheumatoid factor (IgM-RF), as well as high CRP and erythrocyte sedimentation rate (ESR). She was treated with hydroxychloroquine (HCQ) and methotrexate (MTX), plus 10mg of prednisone. She responded very well to the above treatment regimen since last year that developed a disease flare. Since then, adalimumab (ADA), an anti-TNFa inhibitor, was added. This treatment resulted in complete clinical and laboratory remission until recently. Indeed, on September 2, 2020 she experienced low-grade fever (37.7 °C), myalgias, arthralgias, and malaise. Clinical examination revealed no frank arthritis and the laboratory tests showed only a high CRP (14mg/dl - normal values <6) and high ESR (48mm/h). Chest x-ray was normal. Serological tests for viruses and bacteria, as well as blood and urine cultures were negative. However, the test for SARS-CoV-2 was positive. At that time, she was receiving HCQ (200mg/day), MTX (15mg/week), prednisone (2mg/day) and ADA (40mg/14 days subcutaneously) (sc). The last ADA injection was administered a week before the appearance of fever. She was not a smoker. Past medical and family history were unremarkable. We advised her to discontinue MTX and ADA, and to stay in isolation at home according to the national, European and American recommendations.^9–11^ Ten days later, her symptoms gradually subsided, and the symptoms resolved completely after 3 weeks. The repeated test for SARS-CoV-2 was negative. One month later, she was symptom-free, a new test for SARS-CoV-2 was also negative, and she started receiving her immunosuppressive therapy.

## CASE PRESENTATION 2

A 50-year-old man was diagnosed as having RA 4 years ago, on the basis of symmetrical polyarthritis affecting the wrists, elbows and the knees bilaterally, and with positive IgM-RF and high ESR and CRP. He was treated with MTX 20mg/week, plus prednisone 10mg/day. He responded well, but a year later he relapsed, thus tocilizumab (TCZ), an IL-6 receptor antagonist was initiated (162mg/week sc). He responded very well to this treatment regimen, but on September 24, 2020, he complained of arthralgias, myalgias, sore throat, and fever up to 38°C. Clinical examination revealed no frank arthritis, chest x-ray was normal, and laboratory tests showed white blood count of 3.9/10^9^L with normal differential, high acute phase reactants and a SARS-CoV-2 test positive. The rest of the laboratory tests were negative or within normal limits. Past medical and family history were not significant. He was not a smoker. At this time, he was receiving MTX 15mg/week, prednisone 2,5 mg/day and TCZ 162mg/week. The last TCZ injection was administered three days before the initiation of his symptoms. MTX and TCZ were discontinued, and he stayed in isolation at home.^9–11^ A week later, he felt well, without sore throat and fever, and after 2 weeks, he had complete resolution of the SARS-CoV-2 symptoms. The repeated test for SARSCoV-2 was negative and five weeks later a new test for SARS-CoV-2 was also negative so he started receiving the above-mentioned treatment (MTX and TCZ).

## CASE PRESENTATION 3

A 62-year-old female with seronegative RA diagnosed 5 years earlier, was in clinical remission since 2018, receiving HCQ 200mg/day plus Etanercept (ETN) 50mg/week sc. She presented on 18^th^ November 2020 with arthralgias, muscle aches, weakness, sore throat, altered sense of smell, and fever (38.5 °C). She visited our clinic, where after a detailed clinical and laboratory investigation, she was diagnosed as having SARS-CoV-2 infection. She had high ESR and CRP, as well as high ferritin levels (805mcg/L, normal values <35). She remained isolated at home receiving HCQ 200mg/day and azithromycin (AZT) 500mg/day, while ETN was discontinued. She was not receiving other drugs and she was not a smoker. Seven days later, she felt well without fever, sore throat and myalgias, but the olfactory dysfunction was still present. After three weeks she was free of symptoms. The patient started receiving ETN after a new SARS-CoV-2 negative test.

## CASE PRESENTATION 4

A 55-year-old female with seropositive RA since 2016 receiving MTX 20mg/week and golimumab (GLM) 50mg/month sc presented on 22^nd^ November 2020 to us complaining of arthralgias, myalgias, sore throat, and loss of taste and smell. Clinical evaluation revealed no arthritis, while the laboratory tests showed high ESR and CRP, and positive SARS-CoV-2 test. She had no other comorbidities and was not a smoker. She stayed at home receiving some analgesics, while MTX and GLM were discontinued. Two weeks later she felt better, but the loss of taste and smell had no improvement. After one month she was free of symptoms with some improvement of taste and smell. The SARS-CoV-2 test was negative, and she started receiving MTX and GLM.

## CASE PRESENTATION 5

A 38-year-old female with a 12-year history of seronegative RA presented on 15^th^ December 2020 with severe arthralgias and myalgias, as well as fever up to 38.5°C lasting for 3 days. She was on treatment with HCQ 200mg/day and prednisone 2,5mg/day because she delivered a healthy baby 6 months earlier. Past medical and family history were unremarkable. Clinical examination revealed no frank arthritis and she tested positive for SARS-CoV-2. The rest of the laboratory tests showed only high CRP. She continued receiving her drugs plus AZT 500mg/day and she stayed at home. After 10 days she felt better without fever, myalgias and arthralgias. One month later, the test for SARS-CoV-2 was negative.

## DISCUSSION

In **Table 1** we present the clinical and laboratory findings of those five RA patients who were infected with SARSCoV-2. All infected patients presented systemic manifestations and mild upper respiratory symptoms from the nose and throat, while the laboratory investigation revealed only high acute phase reactants, mostly CRP and ESR, while one patient had also high ferritin levels. In addition, one patient had low white blood cells. None of these patients was hospitalized. Cs and bDMARDs were discontinued with the exception of HCQ and prednisone. The outcome of those patients was very good, lasting for 4–5 weeks until complete recovery.

**Table 1.** Clinical and laboratory findings of rheumatoid arthritis patients infected from SARS-CoV-2.

**Patients**	**Systemic manifestations**	**Ear, nose, and throat symptoms**	**Respiratory symptoms**	**Laboratory tests**	**Treatment**	**Outcome**
N:1	Arthralgias, myalgias, malaise, fever	None	None	High ESR High CRP	HCQ 200mg/day, prednisone 2.5mg	Good
N:2	Arthralgias, myalgias, fever	Sore throat	None	High ESR High CRP Low WBC	Prednisone 2.5mg	Good
N:3	Arthralgias, myalgia, weakness, fever	Sore throat, olfactory dysfunction	None	High ESR High CRP High Ferritin	HCQ 200mg/day AZT 500 mg/day	Good
N:4	Arthralgias, myalgias	Sore throat, Loss of taste and smell	None	High ESR High CRP	None	Good
N:5	Arthralgias, Myalgias, fever	None	None	High CRP	HCQ 200mg/day, prednisone 2.5mg/d, AZT 500mg/d	Good

SARS-CoV-2: Severe acute respiratory syndrome coronavirus-2; ESR: Erythrocyte sedimentation rate; CRP: C-reactive protein; WBC: white blood cells; HCQ: hydroxychloroquine; AZT: Azithromycin.

**Figure 1.**
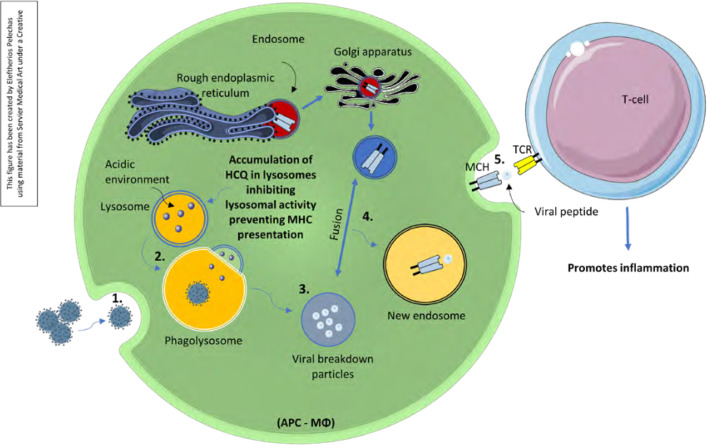
Antigen-processing onto macrophage/APC and the action of hydroxychloroquine. An antigen-presenting cell, takes up the pathogen (SARS-CoV-2) end engulfs it with phagocytosis (1). Lysosomes that contain acids create an acidic environment and fuse with the phagocytosed “invader” creating the phagolysosome which has an acidic environment too (2). Because of the acidic environment, the content of the phagolysosome breaks down leaving various particles that will be processed as antigens (3). In the meantime, within the endoplasmic reticulum the ribosomes are synthesizing MHC and allow the formation of an endosome which passes also through the Golgi apparatus to form a new endosome. The viral breakdown particles will then fuse with the new endosome and the particles will bind onto the groove of the MHC (4). Finally, the antigen presenting cell will express it on its self-surface (5). Hydroxychloroquine, accumulates in lysosomes, raising the pH establishing a non-acidic environment and inhibits the lysosomal activity and, in this way, it prevents the MHC presentation to the surface of the antigen-presenting cell.

Treatment decisions and therapeutic strategies in critically ill patients of SARS-CoV-2 infection were initially limited. Much of the therapy for severe Covid-19 are empirical and depend mostly on clinical judgement.^12^ However, up to date, different therapeutic protocols are applied using anti-viral drugs, steroids (mostly dexamethasone), supportive treatments, and several anti-rheumatic drugs, such as HCQ, cytokine inhibitors, and others.^13,14^ Regarding the patients with ARD infected from SARS-CoV-2, a study from Gianfrancesco et al. showed that patients receiving prednisone >10 mg/day had a higher probability to be hospitalised, while in those receiving anti-TNFa therapy, the risk of hospitalisation was low.^15^ Haberman et al. showed that antimalarial therapy was not associated with the likelihood of hospitalization in patients infected from SARS-CoV-2. On the other hand, bDMARDs and targeted synthetic (ts) DMARDs reduced the odds of hospitalization.^16^ In a subsequent study, the same investigators showed that among patients with ARDs and SARS-CoV-2 infection, the outcome was worse in those receiving corticosteroids, but none in those receiving anti-cytokine therapies.^17^ HCQ has been used in rheumatology for many years, especially for the treatment of RA and systemic lupus erythematosus (SLE). One questions that arises at this point is, how could HCQ help patients suffering from SARS-CoV-2 infection. The answer is simple: we do not know yet, but what we do know is that HCQ inhibits or impairs the presentation process of an antigen, or of a microorganism to macrophages due to inhibition of toll-like receptors (TLRs). Another action of HCQ is that of helping macrophages to destroy the ‘invader’ in the cytoplasm through the action of lysosomes and blocks the major histocompatibility complex (MHC), which is responsible for the presentation of the ‘invader’ from macrophages to T cells. Therefore, T cell activation is inhibited (**Figure 1**). Could the above mechanisms of HCQ block the virus entry, and prevent its multiplication and spreading? We do not know, yet.^13,14^ However, in vitro studies showed that HCQ blocks viral replication by inhibiting the entry of SARS-CoV-2 by interacting with glycosylation of angiotensin converting enzyme-2 (ACE-2) receptor and its binding with the spike protein.^18^ This remains to be documented and there are some trials that are trying to support it.^19–21^ Furthermore, high HCQ blood levels are associated with reduced thrombotic events in SLE patients,^22^ which is also a clinical manifestation of SARS-CoV-2 patients. On the other hand, a study by Koning et al. found no protection for SARS-CoV-2 infection or the development of the severe form of the disease with the use of HCQ in patients with SLE.^23^ Recent data regarding the effect of HCQ in Covid-19 did not show a clear clinical benefit, while a systematic review and meta-analysis demonstrated that HCQ use was not associated with any benefit or harm regarding Covid-19 morbidity.^24^

IL-6, IL-1, and TNFa are the pivotal cytokines responsible for the clinical deterioration of patients suffering from SARS-CoV-2 infection. TCZ is an IL-6 receptor antagonist which is used in rheumatology for patients suffering from RA, juvenile idiopathic arthritis and temporal arteritis. There are several reports, using TCZ for critically ill patients infected with SARS-CoV-2, showing encouraging results.^25–30^ On March 3 2020, TCZ has been approved in China for use in patients with the SARS-CoV-2 infection. Indeed, TCZ maintains a crucial role in critically ill patients with acute progressive SARS-CoV-2. More than 200 cases of SARS-CoV-2 treated with TCZ have been published mostly from Chinese, Italian and Spanish investigators.^31,32^ In almost all cases, TCZ was found to be effective, with an acceptable safety profile. The clinical improvement has been associated with normalization of CRP and other acute phase reactants.^26,28^ In a few studies pulmonary improvement has been also noted by improvement of imaging findings.^30^ On the other hand, other studies, showed that high IL-6 was associated with severe phenotype disease of SARS-CoV-2.^25^ This is probably due to the fact that IL-6 levels may increase a few days after TCZ administration.^33^

Anakinra, is an IL-1 receptor antagonist which is used in RA, Still’s disease, cryopyrin-associated periodic syndrome (CAPS), and in patients with gout. Canakinumab, is a monoclonal antibody against IL-1 and it is used in CAPS and gout patients. Anakinra, showed reduction of CRP and progressive improvement of respiratory parameters in critically ill patients when administered intravenously and in high doses.^34^ Recently, Moutsopoulos HM described a patient suffering from CAPS who received Canakinumab 10 days before of being tested positive for SARS-CoV-2. He reported that the patient had a mild disease course and a week later the test for SARS-CoV-2 was negative.^35^ TNFa inhibitors are used in the last two decades for the treatment of RA, SpAs, and other inflammatory arthritides. It has been shown that the use of TNFa inhibitors in SARS-CoV-2 patients may improve disease outcome and prevent organ damage.^36^ The question that we addressed at the beginning, regarding the impact of SARS-CoV-2 infection in ARD patients, is still a matter of discussion due to limited data so far. However, single reports, as above, case series and observational studies published recently indicated that in patients with ARD and SARS-CoV-2 infection the disease is expressed with mild clinical symptoms without severe consequences probably due to their concomitant use of cs and bDMARDs.^15–17,37–44^

On the other hand, the Covid-19 Global Rheumatology Alliance (GRA) has published a series of papers dealing with the impact of COVID-19 in ARD patients.^45^ The results suggest that as in the general population, age and comorbidities are to blame as the risk factors for poor outcome in ARD patients infected by SARS-CoV-2. Furthermore, glucocorticoids are also associated with an unfavourable outcome in these patients. Finally, Stangfeld et al. who examined the casualties in a large amount of patients with ARDs, found that moderate or high disease activity, and specific drugs (sulfasalazine, azathioprine, cyclosporine-A, mycophenolate mofetil, tacrolimus, cyclophosphamide, and the use of rituximab) were associated with increased odds of COVID-19 deaths.^46^ However, all the above apply to a mixed population of ARD patients (SLE, scleroderma, RA and others) who may have many different systemic manifestations and treatments. The Janus-kinase (JAK) inhibitors tofacitinib (TOFA) and Baricitinib (BARI), have been proposed as potential therapeutic agents for SARS-CoV-2 infection. Especially BARI may block the viral entry into the cells by inhibiting the members of the numb-associated kinase (NAK) family, such as adaptor associated protein-kinase-1 (AAKI) and cyclin-G associated kinase (GAK), which are involved in the viral endocytosis.^47^ Currently, both drugs are used to treat RA and other inflammatory arthritides in a dose of 5mg twice daily for TOFA and 2mg twice daily for BARI. It has been shown that BARI is able to inhibit effectively AAKI and GAK with the above approved dose for RA. Moreover, BARI as a selective inhibitor of JAK 1 and 2 and TOFA as an inhibitor of JAK1 and 3 are also able to inhibit the inflammatory response of SARS-CoV-2, especially IL-1 and INF-γ. Several studies indicated that JAK inhibitors may have some beneficial effect in the treatment of COVID-19.^48,49^ However, recent studies evaluating the impact of the COVID-19 on morbidity and mortality in patients with ARDs indicated that the use of JAK inhibitors had a negative effect.^50,51^

In the present report we present five patients with RA in clinical remission without systemic manifestations and comorbidities who were treated with cs and bDMARDs that achieved a good outcome. Indeed, our RA patients during the SARS-CoV-2 infection were receiving csDMARDs (HCQ and MTX), while three of them were receiving also TNFa inhibitors, and one patient was on treatment with an IL-6 receptor antagonist. All had mild clinical manifestations and very good outcome with complete resolution of their symptoms, without hospitalisation and no additional therapy. Another factor which may have influenced the clinical manifestations of our patients is that none of them had other comorbidities, like diabetes mellitus, coronary disease, hypertension, and other diseases which may play crucial role in the deterioration of the clinical course and outcome of SARS-CoV-2. All patients, except one, restarted their immunosuppressive therapy for RA after a negative test for Covid-19, which is in line with the international guidelines. Alternatively, the immunosuppressive therapy should be restarted after a two-week, symptom free period of the patient.^52,53^ Thus, we can hypothesise that anti-rheumatic drugs may ameliorate the clinical picture and disease course of COVID-19 in RA patients. However, further studies with a large number of patients are needed in order to confirm the above beneficial effects of the anti-rheumatic drugs.
